# Review of Phytochemical Potency as a Natural Anti-*Helicobacter pylori* and Neuroprotective Agent

**DOI:** 10.3390/molecules28207150

**Published:** 2023-10-18

**Authors:** Yohanes Tandoro, Bo-Kai Chen, Asif Ali, Chin-Kun Wang

**Affiliations:** 1 Department of Nutrition, Chung Shan Medical University, 110, Section 1, Jianguo North Road, Taichung 40201, Taiwan; y.tandoro@gmail.com (Y.T.); gargon88@gmail.com (B.-K.C.); asiffstpk@gmail.com (A.A.); 2 Faculty of Agricultural Technology, Widya Mandala Catholic University Surabaya, Surabaya 60265, Indonesia

**Keywords:** phytochemical, neurodegenerative disease, *Helicobacter pylori*, anti-microbial properties, neuroprotection

## Abstract

Phytochemicals are plant secondary metabolites that show health benefits for humans due to their bioactivity. There is a huge variety of phytochemicals that have already been identified, and these compounds can act as antimicrobial and neuroprotection agents. Due to their anti-microbial activity and neuroprotection, several phytochemicals might have the potency to be used as natural therapeutic agents, especially for *Helicobacter pylori* infection and neurodegenerative disease, which have become a global health concern nowadays. According to previous research, there are some connections between *H. pylori* infection and neurodegenerative diseases, especially Alzheimer’s disease. Hence, this comprehensive review examines different kinds of phytochemicals from natural sources as potential therapeutic agents to reduce *H. pylori* infection and improve neurodegenerative disease. An additional large-scale study is needed to establish the connection between *H. pylori* infection and neurodegenerative disease and how phytochemicals could improve this condition.

## 1. Introduction

*Helicobacter pylori* infection is one of the global health problems. More than 50% of the population in the world is affected, mostly in developing countries [1]. *H. pylori* attaches to the human stomach; induces a change in gastric physiology; and is highly associated with gastric ulcers, which further progress into gastric cancer [2]. *H. pylori* can colonize and infect gastric tissue because of virulent factors such as urease, lipopolysaccharide (LPS), vacuolating cytotoxin A (VacA), cytotoxin-associated gene A (CagA), and some others [3]. Until now, the main treatment for *H. pylori* infection is to use the combination of two antibiotics together with a bismuth compound and/or antacid agent such proton pump inhibitor (PPI), which is called quadruple therapy and provides an eradication rate of more than 80% [4]. The usage of antibiotics in *H. pylori* offers another concern of some side effects as well as antibiotic resistance problems [5]. Recent studies show that *H. pylori* infection contributes to the progression of neurodegenerative diseases.

Neurodegenerative diseases (NDs) are disorders that affect the central nervous system and that are mostly caused by neuronal cell death, which causes impairment of the cognitive and motoric system [6]. There are many risk factors associated with ND progression, but its pathogenesis has still been unclear until now. Several diseases are classified as NDs such as Alzheimer’s disease (AD), Parkinson’s disease (PD), and Huntington’s disease (HD) [7]. These diseases have different characteristics, but most of them share the same hallmarks, which are neuronal cell death and neuroinflammation [8,9]. Until now, ND has been classified as an incurable disease, and medication might have a small impact on improving a patient’s condition [10]. Evidence of nutraceuticals on NDs is still deficient, in terms of whether together with normal medication, they could provide better effects on subjects with NDs.

There are several hypotheses about the possible connection between *H. pylori* infection and NDs. *H. pylori* affect the absorption of folate and vitamin B-12, which causes the elevation of homocysteine level and induces neurotoxicity. Furthermore, *H. pylori* cross the blood–brain barrier and induce amyloid deposition in the brain [11]. Another study showed that the outer membrane vesicles of *H. pylori* that were injected into mice altered astrocyte function and induced neuronal damage in the mouse brain [12]. In PD, it showed that *H. pylori* infection is related to the progression of the disease and increases the requirement of medication for PD [13]. This evidence might provide a clue about the connection between neurodegenerative disease and *H. pylori* infection.

Phytochemicals are secondary metabolites of plants, which are non-nutritive bio-active compounds synthesized for natural defenses of the plant against pests [14,15,16,17]. Phytochemicals found in fruits, vegetables, nuts, and grains provide health benefits. Many studies showed that phytochemicals from different natural sources act as antibacterial agents or neuroprotective agents [17,18]. Önem et al. showed that stalk extracts from two different cultivars of *Prunus avium* L. inhibited Gram-positive bacteria and reduced the biofilm formation of bacteria by up to 75% [19]. Li et al. showed that supplementation of proanthocyanidins (PAC)-rich cranberry juice (44 mg of PAC per portion) twice a day for 8 weeks significantly reduced *H. pylori* infection [20]. Desideri et al. reported that high intake (990 mg/day) of dietary flavonols from cocoa for 8 weeks significantly improved cognitive function in mild cognitive impairment subjects compared to those of low intake (45 mg/day) of cocoa flavonols [21]. Kent et al. pointed out that the intervention of anthocyanin-rich cherry juice for 12 weeks significantly improved verbal fluency and short-term and long-term memory in subjects with dementia [22]. Past studies showed that phytochemicals can be used as drug alternatives to treat *H. pylori* and neurodegenerative disease and reduce the risk of antibiotic resistance and complications due to the medication. Hence, this review discusses the potential of phytochemicals from various sources for *H. pylori* infection and also neuroprotection in in vitro and in vivo studies.

## 2. *H. pylori*

**Figure 1:**
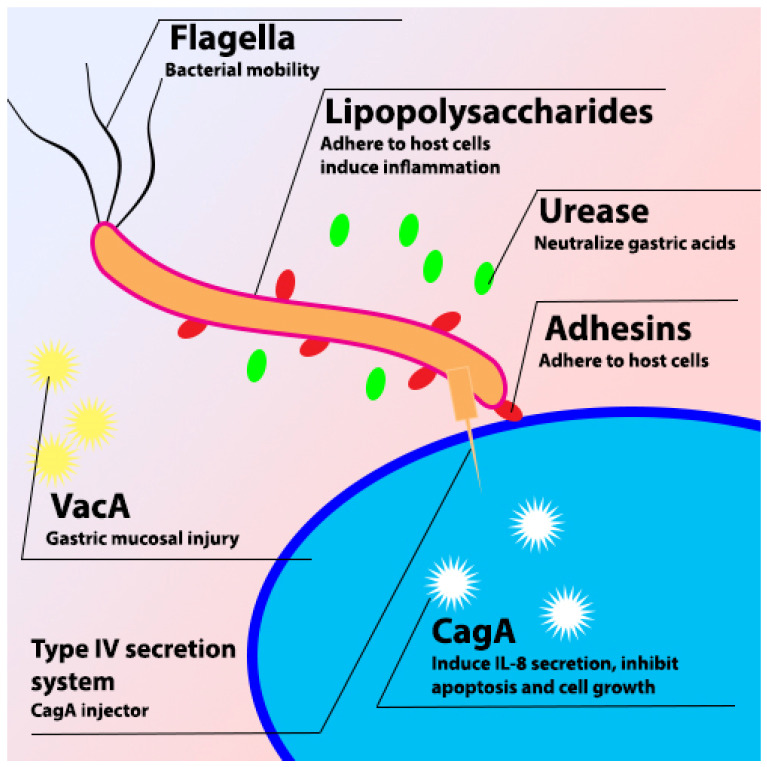
Schematic diagram of *H. pylori* virulence factor.

*H. pylori* is a Gram-negative spiral bacterium that is found in the human stomach and is associated with gastric ulcer and advanced gastric cancer [2,23,24]. The infection of *H. pylori* shows no symptoms in most cases, but it depends on the immune response of the individual and the severity of the syndrome. Most symptoms of *H. pylori* infection are correlated with the gastric ulcer and inflammation in the gastric tissue [25]. *H. pylori* is considered a special bacterium due to the virulence factors (Figure 1) that help to colonize in the human stomach, such as VacA, CagA, urease, LPS, and different kinds of adhesins [3].

### 2.1. VacA

VacA is one of the virulence factors possessed by *H. pylori*. VacA is the major toxic 88 kDa protein that is secreted from *H. pylori* through type V auto transport secretion system (T5SS), which binds to the host cell and causes vacuolation of the cell [26].

VacA plays an important role in the colonization of *H. pylori* in the gastric mucosa, stimulating the autophagy pathway in cells and disrupting lysosomal trafficking that causes the accumulation of dysfunctional autophagosomes and the formation of large intracellular vacuoles to promote the intracellular survival of *H. pylori* [27]. Furthermore, it induces different responses in infected cells such as vacuole formation, cytochrome c release, and forming channels in the mitochondria [28]. It also induces cell apoptosis because of increasing cytochrome c release from mitochondria. Cytochrome c combines with Apaf-1 and caspase-9 to stimulate the production of caspase-3 and caspase-7, resulting in cell apoptosis [29,30]. VacA can disrupt the tight junction to alter the tissue structure and increase the adhesion of *H. pylori* to epithelial cells [26,31,32].

### 2.2. CagA

CagA is a 120 to 145 kDa protein that can be injected into the host cell by using a type IV secretion system (T4SS) after the adhesion of *H. pylori* to the host cell [33]. *H. pylori* is divided into two different strains based on the presence of CagA: CagA-positive and CagA-negative strains. The cagA-positive strain is more virulent than the CagA-negative strain and is associated with higher gastric inflammation [34].

The effects of CagA on the host cell are independent of the phosphorylation process. The most noticeable is to disrupt the cell’s tight junction and induce cell morphology changes [35]. Non-phosphorylated CagA also can activate serum response elements further affect the cell cycle and induce inflammatory response [36].

### 2.3. Urease

Urease is a 550 kDa molecule consisting of UreA and UreB subunits. Urease plays a crucial role in the survival of *H. pylori* in the human stomach. *H. pylori* produces urease in acidic conditions, which breaks down urea and releases ammonia to neutralize the acidic condition in the human stomach [37]. pH increases in the stomach alter the protective mucous layer and also dysregulate the gastric epithelial cell tight junction [38].

### 2.4. Pathophysiology of H. pylori Infection

*H. pylori* infection is associated with chronic gastritis, gastric ulcers, and gastric cancer [1]. Development of gastric problems due to *H. pylori* infection is mostly caused by alteration of the gastric physiology and microenvironment, which induces an immune response from the human body [39]. This immune response is due to the activity of the *H. pylori* virulence factors such as CagA, VacA, and urease, and the response might be different depending on the age [40,41]. Immune response due to *H. pylori* infection is mediated by Toll-like receptors (TLRs) and microRNA, which can promote or suppress the immune response [42]. After reaching the stomach, *H. pylori* move to the mucous layer to evade the acid condition with the help of urease and attach to epithelial cells with the help of different kinds of adhesins such as BabA, SabA, AlpA/B, HopZ, and OipA [43]. After binding to the host cell, *H. pylori* inject different kinds of toxins such as CagA and VacA, depending on the strain, being able to induce inflammatory responses and upregulation of pro-inflammatory cytokines secretion [1].

### 2.5. Diagnosis and Treatment

There are various methods to identify and diagnose *H. pylori*. The invasive tests are based on gastric biopsy and peripheral samples to check the infection of *H. pylori*. On the other hand, the non-invasive method is to use the Urea Breath Test (UBT) C_13_ or C_14_ [1].

UBT is one of the most popular methods to diagnose *H. pylori* infection due to its high sensitivity and is considered the gold standard of the non-invasive method [1]. UBT is based on the reaction of C_13_-labeled urea and bacterial urease secreted from *H. pylori*, which produce ammonia (NH_3_) and C_13_-labeled carbon dioxide in the breath. The concentration of the C_13_ isotope is determined by using gas chromatography and considered positive if the Delta Over Baseline (DOB) value is ≥4‰ [44,45,46].

Treatment of *H. pylori* infection is usually conducted by using antibiotics and combination with PPI and/or with bismuth. Monotherapy (single antibiotic) was used in the past, but the efficacy was poor. The addition of PPI is used as dual therapy in some countries. Overuse of antibiotics induces the mutation and resistance of *H. pylori* and produces some side effects such as dizziness, vomiting, and allergy [47,48].

## 3. NDs

NDs are diseases that occurs in the central nervous system (CNS), being characterized by the progressive reduction of neuronal cells in the brain due to cell death [49]. Until now, there have been no medications that can cure these diseases due to the characteristic of neuronal cells, being unable to regenerate themselves after cell damage and death [50]. The diseases mostly affect elderly people aged >60 years. Recently, it become a public health concern due to them also affecting the younger generation worldwide [51,52,53]. In general, neurodegenerative diseases share a similar major hallmark, which is neuronal cell death, with the major pathways being apoptosis and necrosis with difference and chronic neuroinflammation [8,9]. These conditions could occur due to stress accumulation and misfolded protein deposits, which can induce cytotoxicity events such as impairment of cell signaling, DNA damage, mitochondrial dysfunction, and increased ROS production, which leads to neuronal cell death [8,49,54]. There are various manifestations of NDs, such as AD, PD, HD, and amyotrophic lateral sclerosis [10,50].

## 4. AD

AD is a neurodegenerative disease that causes a decline in cognitive functions and interferes with daily living activities. It is the most common form of dementia, especially for peoples aged over 65 [55]. In the United States, 1 out of 10 peoples at age over 65 years is estimated to suffer from AD, and the prevalence increases with age [56]. The major characteristics of the early stages of this disease are short-term memory loss, including impairment of problem-solving ability, multitasking, and abstract thinking problems. The later stage includes subsequent changes in cognitive ability and behavior [57]. Different stages are classified according to the cognitive impairment degree, including preclinical, mild, and dementia stages [55].

**Figure 2:**
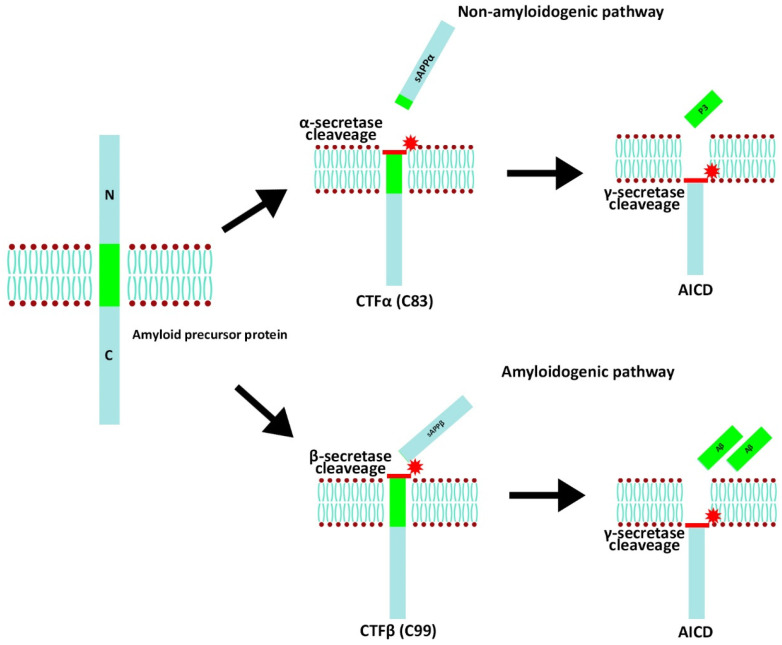
APP proteolytic amyloidogenic and non-amyloidogenic pathway.

AD is a complicated disease. Its initiation and progression into dementia are associated with Aβ and NFT formation [58]. Aβ is a peptide, consisting of 42 amino acids derived from APP [59]. In a normal pathway (Figure 2), APP is cleaved by α-secretase activity producing a large soluble fraction called sAPPα and αCTF, which further cleaved by the activity of γ-secretase, producing AICD and a protein fragment called p83, which rapidly degraded [60]. Aβ is cleaved from APP by β-secretase, and γ-secretase by the amyloidogenic pathway (Figure 2), releasing C terminal peptides that tend to aggregate into oligomers and fibrils to form the senile plaque in the brain [61]. Aggregates of Aβ can cause loss of synaptic plasticity and induce neuronal cell death [62]. The ratio of Aβ 42/40 is a critical point of AD pathogenesis due to its more hydrophobic properties, causing it to be more prone to form oligomers and plaques [60].

Another hallmark in AD is NFT, which is caused by hyperphosphorylation of microtubule-associated protein tau (Mapt) inside the brain, which causes synaptic dysfunction and neuronal loss and leads to dementia [58,63]. Tau is a protein in human neurons that together with tubulin forms microtubules to stabilize the structure of the neuronal cell [64]. Its hyperphosphorylation forms a paired helix filament (PHF) in the brain [65]. On the other hand, hyperphosphorylated tau can bind to normal tau, MAP1, and MAP2 to induce deformation of micro-tubules, causing synaptic and axonal transport dysfunction [66]. Furthermore, insoluble NFT can alter the cytoplasmic function as well as axonal transport in the central nervous system, which leads to neuronal cell death and dementia progression [67].

Until now, AD was categorized as an incurable disease, but some treatments and medication can help to delay the progression [55]. The current treatment is to use cholinesterase inhibitors (ChEI) and partial N-methyl D-aspartate (NMDA) antagonist memantine, which are accepted by the Food and Drugs Administration (FDA) [68]. ChEI was first introduced in 1997 as a medication for mild and moderate AD. There are three types of drugs that are commonly used, namely, donepezil, galantamine, and rivastigmine [69]. ChEI inhibits cholinesterase, which cleaves the neurotransmitter acetylcholine (Ach) and terminates the function [70]. Memantine is an NMDA receptor antagonist that reduces the accumulation of calcium induced by NMDA receptor overstimulation in the neuronal cells [71]. Memantine is often used as a monotherapy or together with a low dose of acetylcholinesterase inhibitor (ACheI) in moderate and severe AD subjects [72]. A combination of memantine and donepezil significantly provides better outcomes on cognition and behavior improvement [73].

## 5. PD

PD is one of the known NDs, with its main features including loss of dopamine-producing neuronal cells in the substantia nigra and some others such as aggregation of α-synuclein protein in neurons and the presence of Lewy bodies in the brain [74,75]. It is characterized by motoric and non-motoric symptoms. The motoric symptoms include bradykinesia, resting tremor, rigidity, and postural instability as well as non-motoric symptoms such as hyposmia, sleep disturbances depression, orthostatic hypotension, constipation, and other dysautonomic symptoms [74,76]. Several risk factors such as age, gender, and ethnicity are associated with PD, but of all these risk factors, age is the greatest risk factor, wherein the prevalence and incidence of PD significantly increases with age [77]. The initiation factor is still unknown, but the progression of this disease is due to the loss of dopaminergic neuronal cells and some factors such as genetic, immune, and environmental factors [76,77]. Dopamine is one of the neurotransmitters that regulates several functions in the brain such as coordinated movement, emotion, and neuroendocrine secretion [78,79].

PD is mostly diagnosed by the presence of bradykinesia with resting tremor and/or rigidity [80]. Bradykinesia is a condition where the speed of spontaneous and repetitive movement is progressively reduced, and this condition usually happens in the early stage [81]. Before bradykinesia occurs in the patient, there is a condition called the prodromal stage, where nonmotor symptoms occur, such as constipation, loss of smell, sleep disorder, and several minor symptoms [82].

Medical therapies are the main treatment for PD, including pharmacotherapy and non-pharmacotherapy [83]. Dopamine receptor or intracerebral dopamine enhancer drugs are the main pharmacotherapies, such as levodopa, dopamine agonist, and monoamine oxidase-B inhibitor [75,83].

## 6. HD

HD is one type of ND that is inherited from parents to their offspring due to the increase of the CAG repeats huntingtin gene on chromosome 4 [84]. The risk of inheritance is equal for men and women, and the carrier’s gene develops the symptoms of this disease in normal life around the age of 40, but the onset of the disease might develop in childhood or teenage years [85,86]. There are several pathogenic mechanisms associated with HD due to the mutation of the Huntingtin protein and causing different brain damage [87]. HD is characterized by striatal degeneration in the brain, loss of medium spiny neurons, and atrophy in different regions of the brain, leading to distinct abnormal movements, psychiatric symptoms, and cognitive deficits that can be fatal 15–20 years after the disease onset [87,88,89].

Up to now, no medication can cure HD, but still, some drug and non-drug treatments can alleviate the symptoms of HD. Drug treatment is used to treat chorea, which is a movement disorder that causes unintended movement in HD patients [90]. Dopamine-reducing drugs such as tetrabenazine and/or antipsychotic agents such as risperidone and aripiprazole are usually used to treat chorea due to HD [84,90]. Non-drug treatment such as physiotherapy might be used to maintain the gait and balance of the subject for a longer period, and psychologists may also help to maintain the mental health of HD patients, which can help to reduce anxiety and depression [86,87].

## 7. Connection between *H. pylori* Infection and Neurodegenerative Diseases

**Figure 3:**
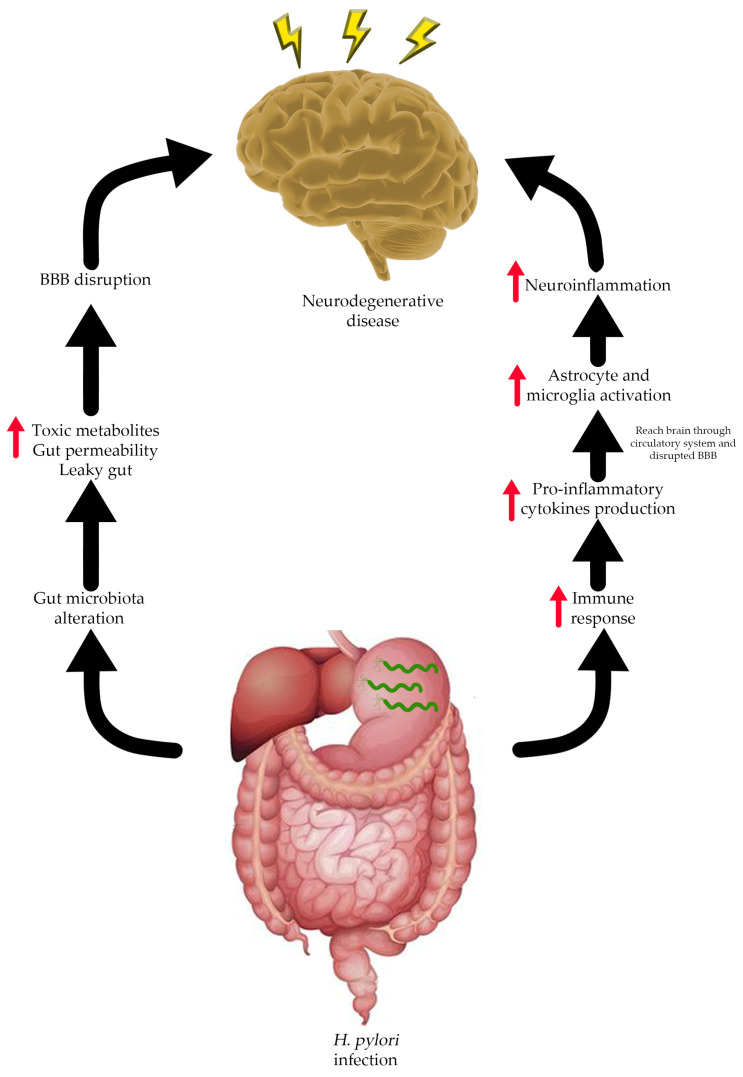
Possible relationship between *H. pylori* infection and neurodegenerative disease.

There are several risk factors correlated with NDs, especially AD, such as age, traumatic head injury, depression, cardiovascular and cerebrovascular disease, and smoking. Recent studies showed that AD is also associated with *H. pylori* infection [91,92]. *H. pylori* is known to infect and cause several health problems in the human gastrointestinal (GI) tract such as gastric ulcers, gastritis, and gastric cancer [93]. In rare cases, a manifestation of extra gastric disease due to *H. pylori* infection might occur with several possible mechanism (Figure 3) and need to be taken into consideration. The extra gastric manifestation due to *H. pylori* infection, especially neurological problems, might occur through alteration of the gut–brain axis (GBA) [94]. The GBA is a bidirectional communication between the central nervous system (CNS) and enteric nervous system that integrates and links the gut and intestinal function with the central nervous system [95,96,97]. GBA modulates the GI function by regulating the GI immune system, mucosal change, and intestinal microbiome in response to stress and emotional and environmental influences [94,98].

*H. pylori* infection is associated with changes in gut microbiome composition [99]. Yang et al. demonstrated that children with gastritis showed an alteration of the gut microbiome, and this condition is worsened by the infection of *H. pylori* [100]. Zheng et al. also showed similar results, wherein in the *H. pylori*-positive subject, the abundance of *Proteobacteria* was increased while the abundances of other phyla such as *Actinobacteria*, *Bacteroidetes*, *Firmicutes*, *Fusobacteria*, *Gemmatimonadetes*, and *Verrucomicrobia* were significantly decreased compared to *H. pylori*-negative subject [101].

Alteration of the gut microbiome composition or so-called gut dysbiosis could lead to increased bacterial amyloid accumulation and intestinal innate immunity response, which induces systemic neuroinflammation, one of the hallmarks of AD [102]. The imbalance of the gut microbiome is related to increased gut permeability and gut barrier dysfunction, which causes toxic metabolites, bile acids, and pro-inflammatory cytokines to enter the circulatory system. The circulating toxic metabolites can reach the CNS and further cause leakage of the blood–brain barrier (BBB) and induce neuroinflammation due to microglia and astrocyte activation [103]. Doulberis et al. propose a hypothesis on how *H. pylori* might directly affect the CNS in three different ways: through the oral–nasal olfactory pathway, blood circulation by infecting monocytes and passing through the disrupted BBB, and the retrograde GI tract neural pathway [104].

Homocysteine (hcy) is one of the sulfur-containing amino acids that is derived from the demethylation process of methionine [105]. Hcy can be further processed into cysteine with the activity of cystathione-β-synthase enzyme and vitamin B6 as a cofactor. This reaction can occur when excess methionine is present in the body. In contrast, when the methionine level is low, hcy can be converted back to methionine by the remethylation process with the help of cofactor vitamin B6 and folic acid [106]. Hcy level in the human body usually ranges around 12–15 μmol/L, and elevation of hcy level is harmful to human bodies. This condition is known as hyperhomocysteinemia [105,107,108], elevated serum hcy is associated with neurological disorders such as cognitive decline, stroke, PD, and AD [109]. This condition can occur due to many factors such as lifestyle, administration of drugs and medication, or diseases such as chronic gastritis [110]. *H. pylori* infection is correlated with gastritis, and this condition can result in deficiency of vitamin B6 and folic acid. Deficiency of these vitamin cause the elevation of serum hcy level [111]. Elevated hcy levels can cause endothelial damage and result in atherothrombotic disorders and progression of AD [112].

Al-baret et al. showed that *H. pylori* infection in C57BL6 WT mice induced neuroinflammation by secretion of pro-inflammatory cytokines in the bloodstream without the deposition of amyloid plaques [113]. AD patients have a higher prevalence of *H. pylori* infection, and *H. pylori* antibodies are found in the cerebrospinal fluid (CSF) of AD patients [92,114]. Roubaud-Baudron et al. showed that *H. pylori*-infected AD subjects were more cognitively impaired and had higher neurodegenerative markers [115]. Wang et al. showed that *H. pylori* filtrate cultured with mouse neuroblastoma N2a cell and injected intraperitoneally into Sprague-Dawley rats induced AD-related tau hyper-phosphorylation in several sites such as Thr205, Thr231, and Ser404, together with the activation of glycogen synthase kinase-3β (GSK-3β) [116]. From the previous study, it might be concluded that *H. pylori* infection and AD might connected due to systemic inflammatory response and also through the gut–brain axis (GBA) interaction.

Apart from AD, PD and *H. pylori* might also correlate with each other through the GBA interaction. Changes in the gut microbiome might affect the metabolite production. As discussed, earlier *H. pylori* infection can induce the growth of Proteobacteria, which consists of mostly known pathogens [117]. Increased growth of pathogens will cause decreased production of short-chain fatty acids and increase the production of bacterial LPS [118]. LPS is the major constituent of the bacterial membrane in Gram-negative bacteria, which is an activator of inflammatory response [119]. LPS is predominantly recognized by Toll-like receptor (TLR) 4, which induces immune response and the release of pro-inflammatory cytokines [120]. *H. pylori* is known to express LPS, and it induces the production of cytokines, which may play a role in the pathogenesis of PD [1,118]. Altered gut microbiome composition also facilitates α-synuclein aggregate migration from the enteric nervous system (ENS) to the brain, causing progression of PD [121]. *H. pylori* infection also affects the absorption of drugs, especially levodopa, due to the change of intragastric pH [122,123].

## 8. Phytochemicals

Phytochemicals are non-nutritive bioactive compounds found in plants [14]. These bioactive compounds are the plant secondary metabolites that show health benefits for humans. Fruits, vegetables, grains, and nuts are the sources of natural phytochemicals. To understand the health benefits of these natural materials, these compounds need to be isolated and identified [124]. Different bioactive compounds show different mechanisms. The combined use of phytochemicals from different sources is needed to achieve greater health benefits. Phytochemicals are divided into several categories as phenolics, alkaloids, saponins, glucosinolates, terpenes, phytoestrogens, nitrogen-containing compounds, organosulfur compounds, carotenoids, and phytosterols [125,126].

### 8.1. Phenolics

Phenolics are one group of plant secondary metabolites consisting of at least one benzene ring and one hydroxyl group, playing important roles in benefitting health [127]. Phenolics can be divided into several subgroups up to the structures [125].

Fruits and vegetables are good sources of phenolics. Dark-colored fruits such as berries contain rich anthocyanins and flavonoids [128]. Cranberry contains 48 different polyphenols consisting of flavan-3-ols, flavonols, anthocyanins, phenolic acid, etc. [129]. Cranberry is rich in A-type proanthocyanidins, which provide health benefits [130]. Black raspberry contains high amounts of phenolics, mostly consisting of anthocyanins and ellagitannins [131]. The anthocyanin contents of black raspberries are the highest when compared with the other rubus species such as red raspberries and blackberry [132].

### 8.2. Carotenoids

There are hundreds of known carotenoids present in nature, but only a few of these carotenoids are good for humans [133]. Past studies showed that consumption of carotenoids was associated with a lower risk of eye problems, cancer, and cardiovascular diseases [134,135,136]. Carotenoids mostly consist of eight isoprenoid units with a total of 40 carbons as the backbone [137]. Carotenoids are divided into two major groups, carotenes (hydrocarbon carotenoids) and xanthophylls (oxygen-containing carotenoids) [138].

Past studies showed that high consumption of carotenoids, especially lycopene, can reduce the risk of cardiovascular diseases by decreasing low-density lipoprotein cholesterol and improving HDL function [139,140,141]. Another study also shows that the consumption of carotenoid-rich products could reduce visceral adiposity and ubiquinol (CoQ10) to prevent metabolic syndrome [142].

### 8.3. Alkaloids

Alkaloids are considered as all-nitrogen-containing compounds aside from peptides and their derivates, amines, cyanogenic glycosides, glucosinolates, cofactors, phytohormones, or primary metabolites [143]. Alkaloids can be classified according to different aspects such as biosynthesis pathways, chemical structure, and taxonomical groups [144].

According to previous studies, alkaloids exhibit different pharmacological activities, such as anti-microbial, anti-cancer, immunomodulatory, and antidiabetic effects [145,146,147,148,149,150].

### 8.4. Saponins

Saponins are bioactive compounds found in a wide variety of plants that are characterized by one or more sugar chains attached to steroid or triterpenoid aglycon backbone [151]. Saponins form foam when agitated in water due to their surface-active properties [152]. Saponins are synthesized from mevalonate primarily in the cytosol via farnesyl diphosphate and squalene [153]. Saponins have a low bioavailability due to their high molecular mass, hydrogen bonding capacity, and molecular flexibility [152].

Past studies have assessed different bioactivity of saponins. They can act as anti-bacterial and anti-fungal agents and act synergistically with antibiotics [154]. Marrelli et al. stated that saponins also exhibit antidiabetic activity by restoring insulin response and increasing insulin secretion from the pancreas [155].

## 9. Effect of Different Phytochemicals on *H. pylori* Infection

**Figure 4:**
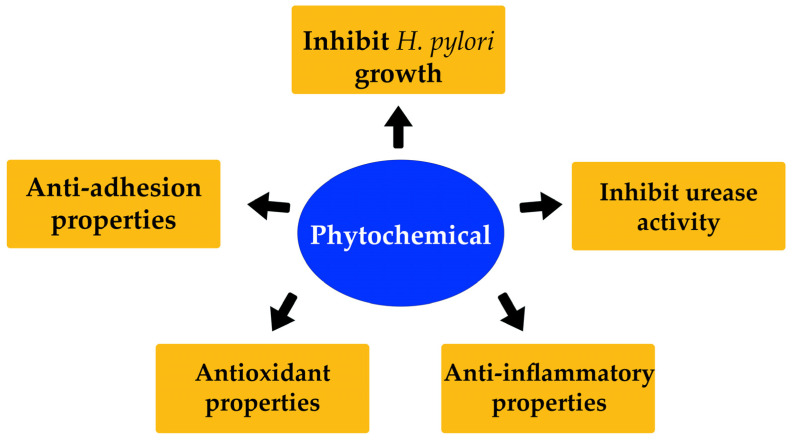
Anti-*H. pylori* activity of phytochemicals from natural sources.

Natural phytochemicals in plants have been already assessed for their potency as anti-*H. pylori* substances (Figure 4). All the natural sources are discussed and summarized in vitro (Table 1) and in vivo (Table 2).

In vitro studies show that most of the phytochemicals reduce the inflammatory response by inhibiting the NF-κB activation and downregulating other pro-inflammatory cytokines [157,158,159]. Gingerol from ginger methanolic extract also inhibits the growth of 19 different strains of *H. pylori*, especially 5 CagA+ strains [156]. Furthermore, ginger shows anti-inflammatory effects and suppresses AP-1 activation [185]. Gaus et al. demonstrated that gingerol from ginger inhibited COX-2, transcription of NF-κB, and release of inflammatory cytokine release in a cell model and animal model [162].

Some other compounds also possess an anti-*H. pylori* effect to reduce the adhesion of *H. pylori* to epithelial cells in both in vitro and in vivo models. Polyphenols, especially flavonoids in monomer and oligomer, show a potential to reduce adhesion of *H. pylori* to epithelial cells [163,164,165,171]. Huang et al. demonstrated the removal of phenolics from noni fruit ethanolic and ethyl acetate extracts causing both extracts to lose antiadhesion activity of *H. pylori* to epithelial cells [143]. Polyphenols from apple peel extract also show the same effect in vitro and in vivo [142].

Cranberry showed anti-*H. pylori* activity due to its A-type proanthocyanidins content [186,187,188]. Gottenland et al. showed that cranberry extract together with *Lactobacillus johnsonii* La1 could reduce *H. pylori* infection. In vivo and in vitro studies show that cranberry can reduce the adhesion of H. pylori to epithelial cells and that A-type proanthocyanidin might play a critical role [24].

Essential oil derived from several herbs also can be used as an anti-*H. pylori* agent. Essential oil from *Dittrichia viscosa* shows potent anti-*H. pylori* activity with the major constituents being 3-methoxy cuminyl isobutyrate, α-cadinol, and α-eudesmol [160]. Another study by Ayoub et al., using *Pimenta racemose* essential oil that contains eugenol, was found to inhibit the growth of *H. pylori* together with inhibition of urease activity [177].

## 10. Effect of Different Phytochemicals on ND Development

**Figure 5:**
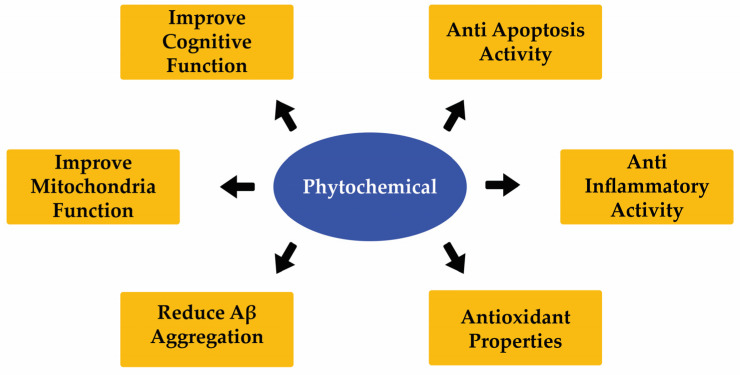
Neuroprotection activity of different phytochemical from natural sources.

NDs were associated with progressive neuronal cell death in the CNS [49]. Until now, there has been no medication to cure ND. Thus, only some medication can improve and delay the symptoms. Recent studies show that some phytochemicals would improve the condition of ND patients. Phytochemicals from natural sources have been assessed to show their potency as neuroprotective agents to improve ND (Figure 5). The neuroprotective activities of natural compounds are presented in Table 3 and Table 4.

Kim et al. and Park et al. showed that different types of curcuminoids, especially calebin-A, curcumin, demethoxycurcumin, bisdemethoxycurcumin, and 1,7-bis(4-hydroxyphenyl)-1-heptene-3,5-dione, can help to protect the PC12 cell line from Aβ insult in vitro, with the effective dose (ED50) ranging from 0.5 to 10.0 µg/mL [178,220]. Yang et al. demonstrated that curcumin can reduce the aggregation of Aβ and also disaggregate fibrillar Aβ40 fragments [221]. Ogunruku et al. found that polyphenol extracts from bell peppers can inhibit the activity of β-secretase (BACE 1) in a dose-dependent manner and also inhibit the aggregation of Aβ40 and reduce fibril formation in vitro [222]. Inflammation is the earliest sign of AD, which is induced by Aβ oligomer through many different receptors such as the Toll-like receptor (TLR) and formyl peptide receptor [223].

Animal studies also showed some phytochemical protection against AD. Purple berry rice contains rich anthocyanins and shows some improvement in AD by preventing memory impairment and hippocampal neurodegeneration in the Wistar rat model [202]. Anthocyanins from purpleberry rice reduces the activity of AChE, which can cleave the neurotransmitter acetylcholine and also reduce lipid peroxidation [70,202]. Another study using aqueous extract of white and red ginger also showed protection against AD in an animal model [203]. Both extracts can inhibit AChE activity and show a synergistic effect on inhibiting AChE activity. Furthermore, it also decreased sodium nitroprusside (SNP) and quinolinic acid (QA) elevated brain malondialdehyde (MDA) content, but there was no significant difference in SNP and QA lipid peroxidation in the brain. Ginsenosides found in ginseng could improve AD subject condition [214,215]. Both studies showed similar results on the improvement of the Alzheimer’s Diseases Assessment Scale (ADAS), Mini-Mental State Examination (MMSE), and Clinical Dementia Rating (CDR) scores in AD subjects. More importantly, results from Lee et al. also showed that after the discontinuation of ginseng powder from the treated group, scores of MMSE and ADAS were declining to the same as the control groups [182]. A high dose (990 mg/day) of cocoa flavonol for 8 weeks could improve the cognitive ability of elderly people with mild cognitive impairment [21].

Phytochemical treatment also provides improvement against other types of ND. Datla et al. demonstrated that treatment of tangeretin, a flavonoid derived from citrus fruit, can protect the neuronal cell from 6-hydroxydopamine (6-OHDA) toxicity in a rat model for PD [198]. Tangeretin can pass through the BBB and protect the dopaminergic neuronal cell, maintain TH+ cells, and significantly increase dopamine levels. Levites et al. demonstrated a positive result of green tea (−)-epigallocatechin-3-gallate (EGCG) treatment on N-methyl-4-phenyl-1,2,3,6-tetrahydropyridine (MPTP)-induced Parkinson’s disease [197]. EGCG treatment protects the neuronal cell against MPTP toxicity, improves antioxidant enzyme activity, and significantly improves tyrosine hydroxylase (TH) activity. TH is an enzyme that plays a role in converting tyrosine into dopamine, and reduced TH activity can cause a reduction of dopamine synthesis and contribute to the progression of PD [224,225].

A previous study also shows improvement in HD by treatment of natural phytochemicals. Sandhir and Mehrotra demonstrated quercetin’s benefit for Huntington’s disease using an animal model, and their result showed that quercetin helps to reverse mitochondrial dysfunction due to 3-nitropropionic acid (3-NP) and reduces mitochondrial oxidative stress [205]. Mitochondrial function is considered to play an important role in the pathogenesis of neurodegenerative disease [226]. Dysfunction of mitochondria can cause alteration in the respiratory chain system, depletion of energy, and increased ROS production, which may lead to the progression of ND [227,228]. Kumar and Kumar also showed that two flavanones derived from citrus fruit, hesperidin and naringin, could protect the neuronal cell and improve mitochondrial function after treatment with 3-NP, and this effect further enhanced the protective effect of hesperidin and naringin when combined with L-NAME, which belong to NOS inhibitor [201].

## 11. *H. pylori* Eradication Improved Cognitive Function in an ND Subject

Until now, the association between *H. pylori* infection and ND is still controversial. Past studies showed that *H. pylori* infection has some common link with ND, especially after the eradication of *H. pylori.* Kountouras et al. showed that *H. pylori* eradication from AD patients showed some improvement in MMSE and Cambridge cognitive test scores compared to subjects who refused the *H. pylori* eradication therapy and *H. pylori*-negative subjects [229]. Furthermore, Chang et al. also showed that *H. pylori* eradication had a linkage with decreased progression of dementia in AD subjects [230]. *H. pylori* infection also interfere with the absorption of antiparkinsonian drugs, which could lead to worsened condition of PD [231]. The evidence obtained from previous studies might provide some research opportunities, especially for phytochemicals to eradicate *H. pylori* infection as well as improve cognitive function.

## 12. Conclusions

Studies showed that phytochemicals from natural sources would be used as anti-*H. pylori* and neuroprotective agents. These compounds can reduce the number of *H. pylori* and alleviate the inflammatory response due to *H. pylori* infection. Natural phytochemicals could be used as a therapeutical agent for *H. pylori* and neurodegenerative disease treatment due to their biological activity and safety concerns. Future studies are needed to find the potential and specific mechanism of each phytochemical in reducing *H. pylori* infection as well as the improvement of ND.

## Figures and Tables

**Table 1:** Assessment of anti-*H. pylori* activity from natural sources in in vitro studies.

Test Material	Activity	Findings	Source
Ginger (Gingerol)	Inhibit *H. pylori* growth	Inhibit growth of CagA+ *H. pylori* strains (MIC: 6.25–50 µg/mL)	[156]
*Curcuma longa* L. (Curcumin)	Anti-inflammatory properties	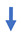 IκBα degradation (up to 80 µM) 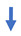 IKKα and β activity (up to 80 µM) 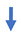 NF-κB DNA-binding (up to 80 µM)	[157]
Chilli pepper (Capsaicin)	Anti-inflammatory properties	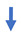 *H. pylori*-induced IL-8 production in MKN45 and AGS cell (100 µM capsaicin, 43.2% and 70%, respectively, compared to control) 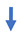 IL-8 mRNA expression (100 µM capsaicin) 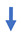 Reduce *H. pylori* NF-κB activation (100 µM capsaicin)	[158]
San-Huang-Xie-Xin-Tang (*Coptis chinesis* Franch, *Scutellaria baicalensis* Georgi, and *Rheum officinale* Baill) (Baicalin)	Anti-inflammatory properties	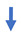 *H. pylori* induced COX-2 enhancement (treatment vs. control group, *p* < 0.05) 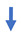 IκBα degradation and nuclear translocation of NF-κB p50 subunit (treatment vs. control group, *p* < 0.05) 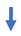 iNOS and IL-8 mRNA expression (treatment vs. control group, *p* < 0.05) 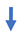 decreased NO and IL-8 production (treatment vs. control group, *p* < 0.05)	[159]
*Dittrichia viscosa* subsp. *Revoluta* (Essential oil (3-methoxy cuminyl isobutyrate, α-cadinol and α-eudesmol)	Inhibit *H. pylori* growth	Essential oil derived from *Dittrichia viscosa* especially fraction 5 and 7 show highest anti-*H. pylori* activity	[160]
Green tea (Catechin and pure sialic acid)	Antioxidant properties	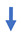 Reduce O_2_^−^, H_2_O_2_ count, NO production (treatment vs control group, *p* < 0.05)	[161]
	Anti-inflammatory properties	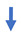 iNOS expression	
	Anti-apoptosis	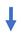 Inhibited apoptosis and reduced apoptosis related protein expression (treatment vs. control group, *p* < 0.05)	
Ginger (Gingerol)	Anti-inflammatory properties	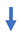 COX-2 (IC_50_: 8.5 µg/mL) 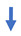 NF-κB transcription (IC_50_: 24.6 µg/mL) 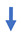 Inflammatory cytokine production (IL-1β, IL-6, IL-8, TNF-α (IC_50_: 3.89, 7.7, 8.5, and 8.37 µg/mL respectively))	[162]
Apple peel polyphenol	Anti-apoptosis	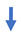 *H. pylori* stimulated vacuolation in HeLa cell (IC_50_: 390 µg GAE/mL)	[163]
	Anti-adhesion properties	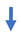 60% adhesion at concentration 5 mg GAE/mL	
*Noni fruit*	Anti-adhesion properties	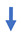 Adhesion of *H. pylori* to AGS cell (treatment vs infected group, *p* < 0.05) 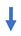 Intracellular CagA level (treatment vs infected group, *p* < 0.05)	[164]
	Anti-inflammatory properties	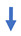 Inflammatory markers (IL-8, iNOS, COX-2) and neutrophil chemotaxis (treatment vs. infected group, *p* < 0.05)	
*Peumus boldus* Mol. (Catechin)	Inhibit urease activity	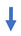 Urease activity from *H. pylori*	[165]
	Anti-adhesion properties	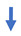 Adhesion ratio of *H. pylori* to AGS cell (treatment vs. infected group, *p* < 0.05)	
*Geranium wilfordii* (Corilagin and 1,2,3,6-tetra-O-galloyl-b-D-glucose)	Inhibit *H. pylori* growth	Ethanol and ethyl acetate extract inhibited *H. pylori* growth (MIC: 40 and 30 μg/mL, respectively)	[166]
*Plantago ovata*	Anti-inflammatory properties	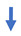 Basal and *H. pylori*-stimulated IL-8 secretion up to 74.51% and 66.67%, respectively (*p* < 0.001) 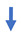 CagA-positive *H pylori*–induced IL-8 mRNA expression up to 67.6% (*p* < 0.0001) 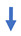 Nf-κB activation (*p* = 0.0001)	[167]
*Bryophyllum pinnatum*	Inhibit *H. pylori* growth	*Bryophyllum pinnatum m*ethanol extract showed anti-*H. pylori* activity (MIC: 32 μg/mL and MBC: 256 μg/mL)	[168]
*Mangiferin indica* (Mangiferin)	Inhibit *H. pylori* growth	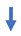 Growth of *H. pylori* (dose dependent, up to 100 μg/mL, *p* < 0.05)	[169]
	Anti-adhesion properties	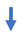 *H. pylori* adhesion to AGS cell (*p* < 0.05, treatment vs. control group)	
	Anti-inflammatory properties	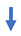 Inflammatory cytokines (NF-κB p65 sub-unit, TNF-α, IL-1β, and IL-8) (*p* < 0.01) and enzyme expression (COX-2, iNOS) (*p* < 0.05)	
*Coptis chinensis Franch* (Berberine, palmatine, coptisine, jatrorrhizine, and epiberberine)	Inhibit *H. pylori* growth	Coptisine showed the highest anti-*H. pylori* activity with MIC and MBC 25 to 50 μg/mL and 37.5 to 125 μg/mL, respectively	[170]
	Inhibit urease activity	Inhibit urease activity and maturation	
Burdock complex *(Arctium lappa*, *Angelica sinensis*, *Lithospermum erythrorhizon*, and *Sesamum indicum* oil)	Anti-adhesion properties	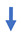 Adhesion of *H. pylori* to AGS cell (*p* < 0.05, compared with *H. pylori*-infected group)	[171]
	Anti-inflammatory properties	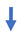 Inflammatory marker (IL-8, TNF-α) (*p* < 0.05, compared with *H. pylori*-infected group)	
Astaxanthin	Antioxidant properties	Prevent the SOD2 level decrease and increase SOD activity, and mitochondrial ROS production in AGS cell	[172]
Blueberry (Cyanindin-3-O-Glucoside)	Anti-inflammatory properties	C3G from blueberry suppressed abnormal DNA synthesis, inflammation, and TLR2 and TLR4 expression; induced apoptosis; and deactivated TLR-mediated NF-κB signaling in LPS-treated cell	[173]
Black raspberry (Anthocyanin)	Inhibit *H. pylori* growth	Inhibited growth of *H. pylori* without having side effects on AGS cell (MIC: 5 µg/mL)	[174]
*Celastrus orbiculatus*	Anti-inflammatory properties	Reduces inflammatory response by regulating epithelial–mesenchymal transition; suppressed methylation of PDCD4 promoter and inhibited microRNA-21, thus enhancing the PDCD4 expression	[175]
*Chrysanthemum indicum* and *Chrysanthemum morifolium* (Essential oil (major constituent camphor))	Inhibit *H. pylori* growth	Both essential oil of *C. indicum* and *C. morifolium* showed potent anti-*H. pylori* activity with IC_50_ 3.63 and 3.78 µg/mL, respectively	[176]
*Pimenta racemosa* (leaves and stem essential oil (eugenol) and methanolic extract)	Inhibit *H. pylori* growth	*Pimenta racemosa* stem essential oil showed the highest anti-*H. pylori* activity compared to others with MIC: 3.9 μg/mL and it inhibited *H. pylori* urease activity simulated with in silico molecular modelling	[177]

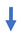
 Indicating decrease in the issue.

**Table 2:** Assessment of anti-*H. pylori* activity from natural sources in in vivo studies.

Test Material	Subject	Activity	Findings	Source
Green tea (Catechin and sialic acid)	Male BALB/c mice	Anti-inflammatory properties	Pre-treatment and post-treatment with catechin and/or sialic acid significantly reduced *H. pylori* infection, mucosal damage, and gastritits score (treatment vs. control group, *p* < 0.05)	[161]
Ginger (Gingerol)	Mongolian gerbils	Anti-inflammatory properties	Significantly reduces mucosal and submucosal inflammation, cryptitis, epithelial degeneration, and erosion due to *H. pylori* infection compared to control	[178]
Polyphenol rich apple peel extract	C57BL6/J mice	Anti-adhesion properties	Administration of apple peel polyphenol could reduce adhesion of *H. pylori*; reduced inflammation, lowering malonaldehyde levels and gastritis score in mice	[163]
		Anti-inflammatory properties		
*Bryophyllum pinnatum*	Swiss mice	Inhibit *H. pylori* growth	*Bryophyllum pinnatum* significantly reduced bacterial colonization in gastric tissue and bacterial load in Swiss mice	[168]
Berberine	Male C57Bl/6 mice	Anti-inflammatory properties	Berberine treatment suppressed pro inflammatory cytokines and upregulated anti-inflammatory cytokines expression	[179]
*Corydalis yanhusuo* (Benzylisoquinoline alkaloids)	Male mice	Inhibit *H. pylori* growth	Two different extracts of *Corydalis yanhusuo* (ethanol and chloroform) inhibited the growth of *H. pylori*, with MIC ranging from 50 to 100 μg/mL and MBC ranging from 100 to 200 μg/mL; chloroform extract of *Corydalis yanhusuo* reduces survival ability of *H. pylori* in gastric mucosa and repairs gastric damage together with reduction of *H. pylori* IgG in infected mice	[180]
Cranberry (A-type proanthocyanidin)	*H. pylori*-positive adults	Anti-adhesion properties	Consumption of cranberry juice could significantly reduce *H. pylori* infection compared to placebo group	[181]
Cranberry (A-type proanthocyanidin)	*H. pylori*-positive adults	Anti-adhesion properties	Cranberry juice addition to standard triple therapy (Omeprazole, Amoxicillin, and Clarithromycin) could significantly improve *H. pylori* eradication rates in female subjects	[182]
Cranberry (A-type proanthocyanidin) and *Lactobacillus johnsonii* La1	Asymptomatic *H. pylori*-positive children	Anti-adhesion properties	Combination of cranberry juice and *L. johnsonii* La1 reduced *H. pylori* infection compared to each test material alone and control group, but no synergistic inhibitory effect observed	[24]
Blueberry and grape seed extract (Proanthocyanidin)	*H. pylori*-positive patient	Antioxidant properties	Combination of blueberry and grape seed extract did not produce a significant change in eradication rate of *H. pylori* compared to placebo group	[183]
Berberine	*H. pylori*-positive patient	Antioxidant properties	No significant difference between berberine containing quadruple therapy eradication rate and adverse effect compared to bismuth containing quadruple therapy	[184]
Burdock complex (*Arctium lappa*, *Angelica sinensis*, *Lithospermum erythrorhizon*, and *Sesamum indicum* oil)	Asymptomatic *H. pylori*-positive subject	Anti-adhesion properties	Significantly reduced UBT value (compared to placebo, *p* < 0.05)	[171]
		Anti-inflammatory properties	Significantly reduced inflammatory marker and (compared to placebo, *p* < 0.05)	
		Antioxidant properties	Improved antioxidant status and plasma phenolic level (compared to placebo, *p* < 0.05) and heal the ulcer in the stomach	
Cranberry (A-type proanthocyanidin)	*H. pylori* positive adults	Anti-adhesion properties	Consumption of high-proanthocyanidin cranberry juice twice a day (44 mg/serving) for 8 weeks could significantly decrease *H. pylori* infection compared to placebo; consumption of encapsulated cranberry powder not significantly effective to reduce *H. pylori* infection	[20]

**Table 3:** Assessment of neuroprotective activity of phytochemical from natural sources in in vitro studies.

Test Material	Cell line	Activity	Findings	Source
*Curcuma longa* L. (Curcumin, demethoxycurcumin, and bisdemethoxycurcumin)	PC12 cells and human umbilical vein endothelial cells (HUVEC)	Anti-apoptosis activity	Three curcuminoids from *Curcuma longa* L. found to protect PC12 cells and HUVEC from Aβ insult	[162]
*Curcuma longa* L. (9 different isolated compounds)	PC12 cells	Anti-apoptosis activity	Five isolated compounds from *Curcuma longa* L. effectively protected PC12 cells from Aβ cytotoxicity	[163]
*Capsicum annuum* var. *grossum* (Polyphenol rich extract)	In vitro study	Reduce Aβ aggregation	Phenolic extract from bell pepper could counteract initial aggregation of Aβ and prevent further aggregation (fibril formation)	[164]
*Bacopa monnieri* (Bacoside-A)	SH-SY5Y cells	Anti-apoptosis activity	Reduced cell cytotoxicity and inhibited fibril formation both in buffer solution only and in the presence of membrane vesicles	[189]
Ginseng (Ginsenoside Rg1)	Primary hippocampal neurons	Anti-inflammatory properties	Ginsenoside Rg1 reduced ROS production, NOX2, and NLRP1 inflammasome due to H_2_O_2_ treatment.	[190]
		Anti-apoptosis activity	Ginsenoside Rg1 also reduced apoptosis, activation of β-galactosidase, and neuronal damage after H_2_O_2_ treatment.	
*Ficus deltoidea* Jack (Vitexin and isovitexin)	Mouse microglial (BV-2) cells	Anti-inflammatory properties	Treatment with *Ficus deltoidea* Jack extract significantly reduced ROS, NO, TNF-α, IL-1β, and IL-6 production from microglial cell after treatment with LPS	[191]
Quercetin	MN9D dopaminergic neuronal cells	Improve mitochondria function	Increased mitochondrial biogenesis and bioenergetics capacity of MN9D cell and reduced 6-hydroxydopmaine induced toxicity	[192]
*Semen ziziphi spinosae* (Jujuboside A)	BV-2 cells	Anti-apoptosis activity	JuA treatment upregulated expression of HSP90β, preserved PPARγ levels, promoted interaction between HSP90β and PPARγ, and promoted the clearance of Aβ42	[193]
*Schisandra chinensis* (Essential oil)	BV-2 cells	Anti-inflammatory properties	*Schisandra chinensis* essential oil treatment decreased NO production and blocked MAPK activation in LPS-stimulated BV-2 microglial cell	[194]
*Dioscoreae nipponicae* (Dioscin)	SH-SY5Y cell	Anti-apoptosis activity	Dioscin improved cell viability	[195]
		Antioxidant activity	Reduce ROS production due to H_2_O_2_ injury in SH-SY5Y cell line	

**Table 4:** Assessment of neuroprotective activity of phytochemical from natural sources in in vivo studies.

Test Material	Compound	Subject	Findings	Source
Ginseng	Ginsenoside Rg3	Male Wistar rats	Ginsenoside Rg3 significantly reduced neuronal apoptosis and apoptosis related protein after treatment of D-galactose; ginsenoside Rg3 also improved antioxidant status and mitochondrial function in D-galactose-induced AD rats	[196]
Green tea extract	(−) Epigallocatechin-3-gallate	Male C57/BL mice	Green tea extract treatment reduced N-methyl-4-phenyl-1,2,3,6-tetrahydropyridine toxicity and prevented dopaminergic neuronal loss	[197]
Citrus	Tangeretin	Male Sprague-Dawley rats	Tangeretin can cross the blood–brain barrier and protect neuronal cells against 6-OHDA toxicity	[198]
	Epigallocatechin gallate	Transgenic mice carrying human G93A mutated SOD1 gene	EGCG treatment prolonged lifespan and the symptoms onset and increased the survival rate of experimental mice	[199]
	Genistein	Male Sprague-Dawley rats	High dose genistein treatment showed neuroprotective effect against 6-OHDA toxicity	[200]
	Hesperidin and naringin	Wistar rats	Pre-treatment with hesperidin and naringin reduced behavioral alteration, oxidative stress, and mitochondrial enzyme dysfunction; this effect was further enhanced when combined with NOS inhibitor (L-NAME)	[201]
*Oryza sativa* (Rice berry, purple)	Anthocyanin	Wistar rats	Prevented memory impairment and hippocampal neurodegeneration; decreased AChE activity and lipid peroxidation	[202]
*Zingiber officinale* (Red and White Ginger)		Wistar strain albino rats	Both extracts inhibited AChE individually and combined together, and both extracts significantly decreased the SNP and QA elevated brain MDA contents	[203]
	Naringin	Male Wistar rats	Improvement of glutathione/oxidized glutathione ratio and reduced free radical level due to 3-nitropropionic acid treatment through Nrf2 activation	[204]
	Quercetin	Female Wistar rats	Quercetin treatment improved mitochondrial function and antioxidant enzymes, as well as reducing astrogliosis and neurobehavioral deficits in experimental rats	[205]
	Genistein	Female Wistar rats	Improvement in Morris water maze result and neuroprotective effect on dopaminergic neuronal cells	[206]
	Quercetin	Albino rats	Significant reduction of behavioral impairment due to rotenone; reduced endoplasmic reticulum stress-induced apoptosis and oxidative stress	[207]
	Quercetin	MitoPark transgenic mice	Improved behavioral change, and reduced dopamine depletion and neuronal loss in MitoPark transgenic mice	[192]
*Momordica charantia*		C57BL/6J and 3 × Tg-AD mice	Prevent memory deficits; reduced neuronal loss, gliosis, Aβ level, and tau hyperphosporylation; and increased synaptic-related protein and pS9-GSK3β expression	[208]
	Chlorogenic acid	Swiss albino male mice	Chlorogenic acid significantly improve motor coordination and antioxidant status. Chlorogenic acid also reduce neuroinflammation and inhibit release of proinflammatory cytokines	[209]
	(−) Epigallocatechin-3-gallate	C57BL/6J mice	Improvement in movement behavior and protection of tyrosine hydroxylase (+) cells against MPTP toxicity, increased CD3^+^/CD4^+^ and CD3^+^/CD8^+^ T lymphocyte ratio, and reduced pro-inflammatory cytokine production	[210]
*Uncaria rhynchophylla*	Isorhynchophylline (IRN)	Male Sprague-Dawley rats	IRN treatment alleviated cognitive decline due to Aβ_25-35_, reduced neuronal apoptosis, and suppressed tau hyperphosphorylation; additionally, IRN also inhibited GSK-3β activity and activated PI3K phosphorylation, which play a role in neuroprotection	[211]
*Semen ziziphi spinosae*	Jujuboside A	APP/PS1 transgenic mice	JuA significantly reduced cognitive deficiency in APP/PS1 transgenic mice, and significantly reduced soluble Aβ42 levels and plaque numbers in the brain	[193]
*Schisandra chinensis*	Essential oil	Male KM mice	*Schisandra chinensis* essential oil can improve cognitive decline in mice, suppressed pro-inflammatory cytokines, and inhibited p38 activation in the mice model	[194]
*Astragalus radix*	Cycloastragenol	C57BL/6N mice	Cycloastragenol upregulated the expression of Nrf2, HO-1, p-TrKB, BDNF, and NeuN and downregulated the expression of p-JNK, p-P-38, and p-Erk; cycloastragenol reduced the activated microglia, inflammatory cytokines, apoptosis, and memory dysfunction	[212]
* Dioscoreae nipponicae *	Dioscin	C57BL/6 mice	Result from in vivo study showed dioscin improved spatial learning and memory; restored MDA, Aβ_42_, AChE, ACh, and SOD levels; and restored brain histopathological change; dioscin downregulated the expression of RAGE and NOX4 and upregulated Nrf2 and HO-1; dioscin also downregulated the levels of p-NF-κB(p-p65)/NF-κB(p65), AP-1, and inflammatory factors	[195]
Citrus		Men and women aged ≥65, living in Ohsaki City, Japan	Frequent consumption of citrus associated with lower risk of getting dementia	[213]
Korean Red Ginseng (KRG)	High KRG dose (9 g/day), low KRG dose (4.5 g/day), control for 12 weeks intervention	61 patients with AD	High dose KRG significantly improved Alzheimer’s Diseases Assessment Scale (ADAS) and Clinical Dementia Rating (CDR) compared to control; KRG group showed improvement on Mini Mental Status Examination (MMSE) but no significant difference with the control group	[214]
Panax Ginseng	Panax Ginseng powder (4.5 g/day) and control for 12 weeks	97 patients with probable AD by NINDS-ADRDA criteria	Baseline MMSE and ADAS showed no difference between 2 groups; after intervention for 12 weeks, the group treated with panax ginseng showed MMSE and ADAS score improvement and after discontinuation of panax ginseng, MMSE and ADAS score declined to the level of the control group	[215]
Cherry juice	Anthocyanin (200 mL of cherry juice/day for 12 weeks)	Elder adult (age 70+) with mild to moderate dementia	Significantly improved verbal fluency, short-term and long-term memory, and reduction of systolic and diastolic blood pressure, but no alteration of inflammation markers	[22]
	Curcumin	Healthy adults	Curcumin administration significantly improved sustained attention and working memory tasks compared to placebo; working memory and mood were significantly better after chronic treatment compared to placebo; curcumin treatment also significantly reduced total and LDL cholesterol	[216]
Cocoa	Flavonol (1 dose daily for 8 weeks) ≈990 mg/day (high), ≈520 mg/day (intermediate) ≈45 mg/day (low)	Elder people with mild cognitive impairment	Time required to complete cognitive and verbal tests was significantly lower in the high and intermediate flavonol groups, compared to the low flavonol group	[21]
Orange Juice	High flavanone (305 mg) and low flavanone (37 mg) daily for 8 weeks	Healthy older adults	High flavanone orange juice gives better improvement on global cognition score compared to the low flavanone group; no significant effect observed of flavanone consumption on mood changes	[217]
	Resveratrol (500 mg/day of Resveratrol (with dose escalation by 500 mg increments every 13 weeks))	People aged > 45 with: Diagnosed with probable AD Mini-Mental State Examination (MMSE) score 14–26 Modified Hachinski Score < 5 stable use of cholinesterase inhibitors or memantine	Resveratrol was safe and well tolerated and some alteration of AD biomarkers were observed but a further and bigger study is needed to find evidence	[218]
Orange juice	Flavonoid-rich orange juice (272 mg/240 mL) or calorie-matched placebo	Males aged 30–65 years old	Flavonoid-rich orange juice improved cognitive function, psychomotor speed, and subjective alertness compared to placebo	[219]
